# Key Points Concerning Amyloid Infectivity and Prion-Like Neuronal Invasion

**DOI:** 10.3389/fnmol.2016.00029

**Published:** 2016-04-22

**Authors:** Alba Espargaró, Maria Antònia Busquets, Joan Estelrich, Raimon Sabate

**Affiliations:** Faculty of Pharmacy, Laboratory of Conformational Diseases, Department of Physical Chemistry, Institute of Nanoscience and Nanotechnology, University of BarcelonaBarcelona, Spain

**Keywords:** Alzheimer’s disease, amyloid, amyloid cytotoxicity, amyloid transmission, Creutzfeldt-Jakob disease, prion, transmissible spongiform encephalopathy

## Abstract

Amyloid aggregation has been related to an increasing number of human illnesses, from Alzheimer’s and Parkinson’s diseases (AD/PD) to Creutzfeldt-Jakob disease. Commonly, only prions have been considered as infectious agents with a high capacity of propagation. However, recent publications have shown that many amyloid proteins, including amyloid β-peptide, α-synuclein (α-syn) and tau protein, also propagate in a “prion-like” manner. Meanwhile, no link between propagation of pathological proteins and neurotoxicity has been demonstrated. The extremely low infectivity under natural conditions of most non-prion amyloids is far below the capacity to spread exhibited by prions. Nonetheless, it is important to elucidate the key factors that cause non-prion amyloids to become infectious agents. In recent years, important advances in our understanding of the amyloid processes of amyloid-like proteins and unrelated prions (i.e., yeast and fungal prions) have yielded essential information that can shed light on the prion phenomenon in mammals and humans. As shown in this review, recent evidence suggests that there are key factors that could dramatically modulate the prion capacity of proteins in the amyloid conformation. The concentration of nuclei, the presence of oligomers, and the toxicity, resistance and localization of these aggregates could all be key factors affecting their spread. In short, those factors that favor the high concentration of extracellular nuclei or oligomers, characterized by small size, with a low toxicity could dramatically increase prion propensity; whereas low concentrations of highly toxic intracellular amyloids, with a large size, would effectively prevent infectivity.

## Introduction

Many neurodegenerative diseases are characterized by the aggregation of misfolded proteins in the brain, the so-called amyloids. Among these disorders are the prion diseases (Creutzfeld-Jakob disease in humans, bovine spongiform encephalopathy in cattle or scrapie in sheep) and non-prion diseases (Alzheimer’s disease (AD), Parkinson’s disease (PD) or tauopathies). The presence of amyloids in neurodegenerative diseases appears to be a truly generic phenomenon. As a differential property, prion diseases are assumed to be transmissible whereas non-prion diseases are non-transmissible.

In prion diseases, through a polymerization process, the misfolded proteins (the prions) become a self-perpetuating infectious agent. In this way, they can become neurotoxic elements in mammals or protein-based genetic elements in fungi (Chien et al., 2004; Aguzzi and Calella, 2009). In mammals, the central event in prion pathogenesis is the conformational conversion of the normal host prion protein (PrP^C^) into an abnormal protease-resistant form (PrP^Sc^) associated with disease. PrP^Sc^ propagates by imposing its abnormal conformation on other PrP^C^ molecules. But this does not explain how infectious prions proceed to induce the spongy brain lesions of transmissible spongiform encephalopathies and, eventually, extensive neuronal death (Aguzzi, 2005; Chesebro et al., 2005). Hence, although the presence of PrP^Sc^ has generally been assumed to be constitutional of neurotoxicity, at the present, PrP^Sc^ itself is considered innocuous. In conclusion, the dissociation of the toxic species (what actually kills neurons) and infectious agent (the propagating PrP) has been well established (Hill and Collinge, 2003a,b; Mallucci et al., 2003; Sandberg et al., 2011). Meanwhile, most prion infectious agents are relatively species specific, but cross-species transmissions have occurred in nature and in laboratory experiments (Race et al., 2015).

In non-prion diseases, a host-derived protein is misfolded and persists in an aggregated form that may damage nearby cells [β-amyloid (Aβ) in AD, α-synuclein (α-syn) in PD, and tau-protein (τ) in tauopathies and AD (Costanzo and Zurzolo, 2013; Goedert, 2015)]. Although prions and amyloids related to non-prion diseases share structural properties and their conformation, only a small handful of non-prion amyloids display the main prion behavior, i.e., the capacity to spread the self-propagating misfolded proteins from neuron to neuron throughout the brain. In recent years, evidence for prion-like mechanisms in neurodegenerative diseases has come to light. Thus, proteins such as τ (Polymenidou and Cleveland, 2012; Walker et al., 2013; Goedert et al., 2014; Holmes and Diamond, 2014; Hyman, 2014; Clavaguera et al., 2015; Polanco and Götz, 2015), α-syn (Hansen et al., 2011; Freundt et al., 2012; Polymenidou and Cleveland, 2012; Spillantini and Goedert, 2013; Goedert et al., 2014; Reyes et al., 2014; Herva and Spillantini, 2015), Aβ (Bahr et al., 1998; Kane et al., 2000; Meyer-Luehmann et al., 2006; Eisele et al., 2010; Münch et al., 2011; Nath et al., 2012; Stöhr et al., 2012; Walker et al., 2013), huntingtin (Ren et al., 2009; Trevino et al., 2012; Banez-Coronel et al., 2015), SOD1 (Münch et al., 2011; Polymenidou and Cleveland, 2011) or TDP-43 (Furukawa et al., 2011) have been shown to undergo seeding aggregation in cell cultures, and some of them even exhibit trans-cellular propagation and the induced spread of pathology *in vivo* (Goedert et al., 2010; Kaufman and Diamond, 2013). That the conformational changes undergone by τ, Aβ, and α-syn could spread between cells was first established in cell models (Petkova et al., 2005; Desplats et al., 2009; Frost et al., 2009). Like classical prions, these proteins form distinct conformers *in vivo*, and Aβ, mutant τ, and mutant α-syn can cause the spread of regional pathology and disease progression in mouse models (Aguzzi and Rajendran, 2009; Clavaguera et al., 2009; Jucker and Walker, 2011; Luk et al., 2012; Mougenot et al., 2012; Stöhr et al., 2012). Moreover, the propagation and misfolding of wild type α-syn have also been reported (Luk et al., 2012; Masuda-Suzukake et al., 2013); although it has never been shown to invade neurons. Neuronal invasion entails dissemination through the peripheral (spleen) and central nervous system (CNS) via distal neuronal spreading, as well as individual-to-individual infection under natural conditions. Thus, the capacity of non-prion amyloids to spread is limited to neuron-to-neuron transmission (Desplats et al., 2009; Freundt et al., 2012; Soto, 2012; Reyes et al., 2014). As in prions, the spread of misfolded protein in non-prion amyloids is not evidence of neurodegeneration (Halliday et al., 2014). That is, misfolded proteins in non-prion amyloids can propagate in a “prion-like” manner, but that spreading is, at least partly, separate from neurotoxicity.

**Figure 1:**
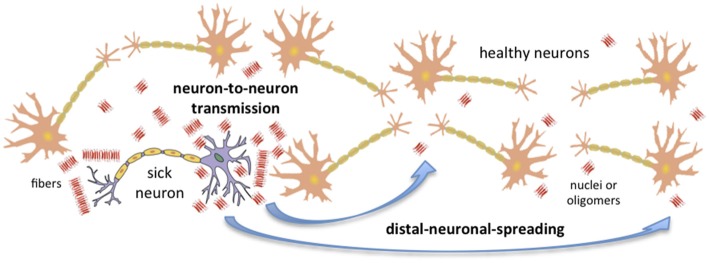
**Amyloid transmission mechanisms.** Amyloid aggregates could be transmitted from a sick neuron to healthy neurons via two main mechanisms: neuron-to-neuron transmission and distal neuronal spreading. The concentration of nuclei, the presence of oligomers, and the toxicity, resistance and localization of these aggregates are key factors affecting putative neuronal invasion. Note that in order to simplify the figure, only neurons have been considered; nonetheless, it is important to take into account that astrocytes and other cells can also sporadically generate aggregates.

Recent advances in the field of conformational diseases, and especially in our understanding of unrelated PrP prions (i.e., yeast and fungal prions), have shed some light on the key factors that determine the capacity of any amyloid to spread. We now have some indication of which factors limit or favor neuron-to-neuron transmission and distal neuronal spreading (Figure 1). In mammals and humans, the prion-like capacity to spread within an individual seems to be intimately linked to neuronal invasion and ultimately to the individual-to-individual infective capacity.

This review covers the prion-like mechanisms of propagation involved in the spreading of the amyloid conformation. Two potential mechanisms for spread within an individual infected organism (intra-individual spreading) are proposed: (1) neuron-to-neuron transmission, referring to the infection of healthy neighboring neurons directly connected to the infected one; and (2) distal neuronal spreading, entailing the infection of healthy neurons located far from the infected one. Moreover, this review attempts to elucidate, in a simplified form, the key factors that favor or impede neuron-to-neuron transmission and distal neuronal spreading, and thus the neuronal invasion capacity and individual-to-individual infectivity of non-prion amyloids. However, we are aware that other potential mechanisms, involving cell types other than neurons, may also be involved in amyloids spreading. Thus, direct crossing of the blood barrier (Banks et al., 2009), dissemination via astrocytes and fibroblasts (Hollister et al., 2015), infection by inducing microglia recruitment (Baker et al., 2002; Marella and Chabry, 2004; Pearce et al., 2015; Tu et al., 2015) or spreading via immune system cells (Isaacs et al., 2006; Bradford and Mabbott, 2012) represent other potential spreading mechanisms.

## Number of Amassed Nuclei

Amyloid fibrillogenesis is a nucleation-dependent process which depends on protein concentration and can be promoted or triggered by homologous preformed amyloids that act as templates in a mechanism known as seeding (Jarrett and Lansbury, 1993; Chiti and Dobson, 2006). The simplified nucleation–elongation model is divided into three phases: (1) the lag phase, when the soluble and monomeric species cluster to form nuclei; (2) the elongation phase, when monomeric species are exponentially added to previously formed nuclei, entailing the formation of transient species such as protofilaments and protofibrils; and (3) the maturation phase, when the transient species as well as oligomers are grouped together, leading to fibril maturation (Jarrett and Lansbury, 1993). However, in a more realistic scenario, amyloid aggregation has to be considered as an inter-conversional multi-step equilibrium of numerous conformational states, which involves a complex network of equilibriums, wherein secondary nucleation and fragmentation events are also possible (Chiti and Dobson, 2006; Knowles et al., 2009; Cohen et al., 2013; Meisl et al., 2014; Sabate, 2014). *In vivo*, seeds can trigger amyloid aggregation, promoting the rapid development of symptoms and increasing the infective capacity of amyloid-prone proteins (Aguzzi and Calella, 2009; Stöhr et al., 2012). Thus, the seeding capacity (which is an intrinsic property shared by all amyloids) of yeast and fungal prions has been shown *in vivo* to be intimately related to the number of nuclei of aggregation per cell as well as the number of amassed nuclei introduced into the cell, which finally determines the probability of transmission of the prion (Maddelein et al., 2002; Tanaka et al., 2006). No relationship has been demonstrated between seeding capacity and the number of nuclei introduced into the cell in the case of mammal prions. However, as seeding is a feature shared by all amyloids, it would seem logical to extrapolate it to the PrP case. Moreover and very interestingly, the possibility of a direct relationship between the concentration of nuclei and the amount of oligomeric material, via “nucleation growth”, has been proposed (Lomakin et al., 1996, 1997; Ferrone, 1999; Serio et al., 2000; Morris et al., 2008; Bemporad and Chiti, 2012). This suggests that the seeding capacity would be dependent on the concentration of oligomers (Sakono and Zako, 2010).

In amyloid aggregation, the final concentration of each amyloid species is highly dependent on both intrinsic and extrinsic factors. Consequently, modification of the factors involved in aggregation changes the nucleation and elongation rates, as well as secondary nucleation or fibril fragmentation. Moreover, such changes would imply subtle alterations in the delicate network of equilibriums. It has been shown in fungal prions that the fragmentation of the chaperone Hsp104 increases the number of nuclei per cell; such an increment is essential for prion transmission (Uptain and Lindquist, 2002; Malato et al., 2007). In short, whereas the amino acid sequence of any amyloid-prone protein can determine the conformation and concentration of amyloid species in the network, changes in the cellular conditions (for instance, as a consequence of stress) could alter the inter-equilibrium among amyloid states, resulting in changes in the concentration of nuclei. It should further be taken into account that specific mutations in the primary protein sequence could alter the aggregation capacity of amyloid-prone proteins, entailing changes in the ratios between aggregate states, which could drastically modify infective capacity (Chiti and Dobson, 2006; Aguzzi and Calella, 2009). As mentioned above, seeding capacity is directly related to the number of seeds (also termed “events” or “propagons” in yeast and fungal prions) per cell. Furthermore, since seeding is essential for amyloid transformation, the number of seeds per cell could become a crucial factor in the amyloid self-assembly process and in their later propagation. Thus, an increment in the number of amyloid-like aggregates must favor neuron-to-neuron transmission.

## Aggregate Size and Spreading Capacity

It is widely accepted that both intrinsic structural characteristics and the size of the species usually formed at the early stages of amyloid aggregation, i.e., oligomers, protofibrils, protofilaments and nuclei (amyloid-like species), can be factors that determine the capacity to spread. Moreover, it has been stated that small amyloid-like species spread the most (Chiti and Dobson, 2006; Aguzzi and Calella, 2009; Nath et al., 2012; Figure 1). If we consider a fixed mass of protein, the size of the aggregates is inversely proportional to the number of seeds per cell, since more aggregates of discrete size would have to be formed to transform soluble protein in its native state into amyloid. Because small aggregates are the most dispersible amyloid-like species, an increased number of these favors both neuron-to-neuron transmission and distal neuronal spreading. For instance, PrP^Sc^ oligomers, composed of 14–28 monomers, have been shown to be the most infectious PrP particles (Silveira et al., 2005).

## Toxicity of Amyloid Aggregates

The *in vivo* cytotoxicity derived from amyloid aggregation is a consequence of an extremely complex and poorly understood combination of interrelated processes. Among other effects, it eventually entails the formation of pores, homeostatic dysfunction and membrane disruption; which seem to be effects induced by many amyloids (Lashuel et al., 2002; Chiti and Dobson, 2006; Lashuel and Lansbury, 2006). In addition, increasing evidence suggests that cell membranes are potential targets of amyloid aggregates and where much of the molecular damage they cause occurs (Walker et al., 2006). Interactions of amyloid aggregates with phospholipid membranes resulting in lipid peroxidation as well as intracellular aggregation leading to the impairment of cellular functions are believed to be potential causes of later cell death (Walker et al., 2006). Crucially, evidence increasingly indicates that the inherent cytotoxicity of amyloids depends of the amyloid state (Lashuel et al., 2002; Chiti and Dobson, 2006; Lashuel and Lansbury, 2006). Thus, oligomers are usually proposed as one of the most toxic species (Ono et al., 2009; Bemporad and Chiti, 2012). It has been suggested that small amyloid-like aggregates, ranging from 5 to 30 monomers, and to a lesser extent mature fibers could principally be responsible for amyloid toxicity and prion infectivity (Prangkio et al., 2012). The debate concerning the relationship between infectivity and neuronal degeneration is far from resolved; but recent evidence from fungal prions shows that the presence of extremely toxic oligomeric species fully prevents prion propagation (Greenwald et al., 2010; Mathur et al., 2012; Seuring et al., 2012). In summary, for aggregates of the same size, higher toxicity seems to be associated with lower infectivity; while less toxic aggregates would be the most infectious (Sabate, 2014). For instance, it could be speculated that *in vivo* PrP^Sc^ aggregates, which are highly infectious, possess low toxicity and that they might not even be toxic particles *per se* (Silveira et al., 2005; Chiti and Dobson, 2006; Aguzzi and Calella, 2009). In contrast, *in vitro* PrP^Sc^ oligomers, sometimes not considered to be bona fide authentic PrP^Sc^, display extreme toxicity and lack infectivity (Simoneau et al., 2007). In the same way, Aβ aggregates, widely accepted as displaying high toxicity, have largely been considered as non-infectious (Canevari et al., 2004; Chiti and Dobson, 2006; Aguzzi and Calella, 2009). Although certain infectivity under forced conditions in primates and transgenic mice (e.g., by intra-cerebral inoculation) has been observed in recent years (Walker et al., 2006), infection under natural conditions has never been demonstrated.

Interestingly, toxicity could exert a dual effect on the infective capacity of amyloids. Intracellular amyloids, which have to diffuse from the neuron to the extracellular matrix, require both passive and active mechanisms (Visanji et al., 2013). On the one hand, neuron death, entailing a lack of active mechanisms for amyloid diffusion, could act as a limiting factor in amyloid distribution. Nevertheless, it is important to take into account that, in certain cases, toxicity could also provoke membrane disruption thereby favoring the opposite effect: the extracellular release of aggregation nuclei. Thus, toxic aggregates may kill cells and thereby be freely released into the medium, increasing their chances of spreading. On the other hand, neuron apoptosis and death entails the inhibition of cellular protein production and hence a reduction in the putative amyloid fibrillogenesis, affecting both intracellular and extracellular amyloids. Consequently, high amyloid toxicity involves accelerated neuron death and consequently a premature end to the amyloid process. Since under these conditions self-polymerization is aborted in the early stages, the final amount of aggregated protein will tend to be extremely low, thereby drastically reducing infectivity. These concomitant processes greatly limit potential amyloid dissemination from the neuron to the extracellular matrix, and the number of nuclei per neuron. Thus, while amyloid toxicity is responsible for neurological damage, high toxicity levels would reduce the capacity of the amyloid to spread.

## Amyloid Resistance

High resistance to denaturation could be considered another generic intrinsic characteristic of amyloids and prions. Infectious PrP^Sc^ displays the relevant physical, chemical and enzymatic resistance (Sabate, 2014), entailing insufficient cellular clearance, bioavailability, transport and spreading (Aguzzi and Calella, 2009; Soto, 2012; Domert et al., 2014). Since PrP^Sc^ cannot easily be degraded by neuronal proteases, the infectious protein persists in the cell, potentiating the seeding capacity and distribution possibilities, and thereby amplifying the infection process.

Importantly, although not yet proven in mammal and human prions, fibril resistance to denaturation could be a key factor in the fragmentation of amyloids. Thus, high resistance has typically been associated with increased fibril rigidity in yeast prions (Tanaka et al., 2004; Castro et al., 2011); this in turn is linked to fragmentation capacity. In this way, more rigid amyloids are not effectively fragmented and generate seeds less efficiently. Since all amyloids appear to show enclosed features, the possibility that a high rate of fibril fragmentation would to be required to favor the spreading of prions would have to be taken into account for all amyloids, including PrP. In summary, the presence of brittle amyloid fibrils increases the number of aggregation nuclei and thereby promotes prion spreading.

## Amyloid Location

Amyloid location could be a key factor for later spreading. We can envisage two different scenarios: the spreading of extracellular (e.g., Aβ and PrP^Sc^) or intracellular (e.g., α-syn, τ or huntingtin protein) amyloids (Sabate, 2014). In the extracellular case, the aggregation process occurs on the extracellular membrane surface, and directly enables both neuron-to-neuron transmission and distal neuronal spreading. In contrast, in intracellular amyloids, the aggregation process occurs in the cytosol or nucleus of the neurons, meaning that potential seeds have to be: (1) leaked from an injured neuron to the extracellular matrix; and then (2) internalized from the external matrix into healthy neurons via passive or active mechanisms (i.e., including transference via exocytosis and endocytosis, accumulation into exosomes or micro-vesicles, tunneling nanotubes, tubular membrane bridges interconnecting neurons, and direct synaptic contact; Sherer and Mothes, 2008; Aguzzi and Calella, 2009; Emmanouilidou et al., 2010; Visanji et al., 2013; Narkiewicz et al., 2014). Importantly, whereas neuron-to-neuron transmission could occur via both passive and active mechanisms, distal neuronal spreading is limited to active secretory processes via exocytosis–endocytosis. This suggests that cell death, implying the suppression of active secretion mechanisms, could be extremely unfavorable for spreading; in particular for intracellular amyloids. Although increasing evidence suggests the presence of intracellular amyloids in extracellular biological fluids such as the cerebrospinal fluid, human plasma or saliva (El-Agnaf et al., 2003; Arnoys and Wang, 2007; Devic et al., 2011; Visanji et al., 2013), the concentration of these amyloid-like aggregates in extracellular fluids is very limited. Interestingly, this concentration is expected to be higher in the inter-neuronal space. This would suggest that, although drastically limited, neuron-to-neuron transmission would still be possible, whereas the distal neuronal spreading would be highly improbable.

## Concluding Remarks

As sketched here, infective capacity could be considered a generic property shared by all amyloids. However, there are several factors that modify the possibility of a non-prion amyloid exhibiting all the properties of a prion. In brief, those factors that favor a high concentration of extracellular nuclei of low toxicity, characterized by a limited size, could dramatically increase prion propensity; whereas, a low concentration of highly toxic large intracellular amyloids would prevent infectivity. Consequently, in humans and mammals, although several amyloid-prone proteins can display certain neuron-to-neuron transmission, distal neuronal spreading as well as other potential distribution mechanisms involving not only neurons (e.g., direct crossing of the blood brain barrier or via microglia and immune system cells) required for individual-to-individual infection, have proven to be insufficient to provoke prion activity under natural conditions. Thus, individual-to-individual infection currently seems to remain restricted to PrP.

## Author Contributions

AE, MAB and JE collaborated in the drafting of the manuscript and RS supervised and drafted the manuscript and edited the draft.

## Conflict of Interest Statement

The authors declare that the research was conducted in the absence of any commercial or financial relationships that could be construed as a potential conflict of interest. The reviewer NS and handling Editor declared their shared affiliation, and the handling Editor states that the process nevertheless met the standards of a fair and objective review.
